# PHACE Syndrome Presenting With Retinal Degeneration, Cortical Dysplasia, Microphthalmia, and Atrial Septal Defect in a South Asian Boy

**DOI:** 10.7759/cureus.12928

**Published:** 2021-01-26

**Authors:** Kavinda Dayasiri, Vijayakumary Thadchanamoorthy

**Affiliations:** 1 Paediatrics, Base Hospital Mahaoya, Mahaoya, LKA; 2 Clinical Sciences, Faculty of Health Care Sciences, Eastern University, Batticaloa, LKA

**Keywords:** phace syndrome, microphthalmia, cortical dysplasia, boy

## Abstract

PHACE syndrome is a rare disorder of vasculogenesis that occurs during the first trimester of pregnancy. The disorder commonly presents with posterior fossa brain anomalies and coarctation of aorta/arterial anomalies and is predominantly seen in female patients. Herein, we report a male child with PHACE syndrome who had several less common features such as cortical dysplasia, retinal degeneration, and microphthalmia. The diagnosis of PHACE syndrome was confirmed based on revised diagnostic criteria and by the presence of one major criterion and two minor criteria in addition to >5 cm haemangioma. Further, the reported child had atrial septal defect as the only cardiac abnormality, and this has been rarely described in patients with PHACE syndrome.

## Introduction

PHACE syndrome is a rare neurocutaneous disorder associated with extensive infantile haemangiomas with approximately 300 cases reported to date [1]. The acronym PHACE stands for posterior fossa anomalies, haemangioma, arterial anomalies, coarctation of aorta/cardiac anomalies, and eye anomalies. The constellation of these clinical abnormalities was first coined as a distinctive syndrome in 1996 [2]. However, following a number of reported cases, the initial collection of specific clinical conditions was transformed to more objective criteria to aid diagnosis. The diagnostic criteria for PHACE syndrome were first established in 2009 based on structural and vascular involvement in the brain, eyes, midline structures, and cardiovascular system [3]. The revised diagnosis and care recommendations published in 2016 [4] updated the previous diagnostic criteria. The recommendations further proposed key actions to improve health surveillance and detect comorbidities early. PHACE syndrome has been rarely reported in male children, with 90% of reported patients being female (1). Clinical features and their severity are highly variable in reported cases, with more common features being posterior fossa brain abnormalities, arterial anomalies/coarctation of the aorta, and migraine-like headaches [4].

The authors report the case of a South Asian boy in whom the diagnosis of PHACE syndrome was confirmed based on the revised diagnostic criteria and who presented with several less common and atypical features of PHACE syndrome.

## Case presentation

A 3.5-year-old boy was referred for evaluation of developmental delay. The parents first became concerned about his development at the age of two years. He was born to non-consanguineous parents with no family history of neurological disorders. Pregnancy, labor, and delivery were uncomplicated. The patient had an older sibling with no developmental delays or other illnesses. He had had larger, reddish, soft plaques over his left upper face and right upper back, which had reduced in size over time. The lesions had been present at birth as tiny papules and subsequently, they increased in size over the first two years. Developmental assessment at 3.5 years revealed a predominant delay in speech, communication, and fine motor skills with borderline delay in gross motor skills.

**Figure 1:**
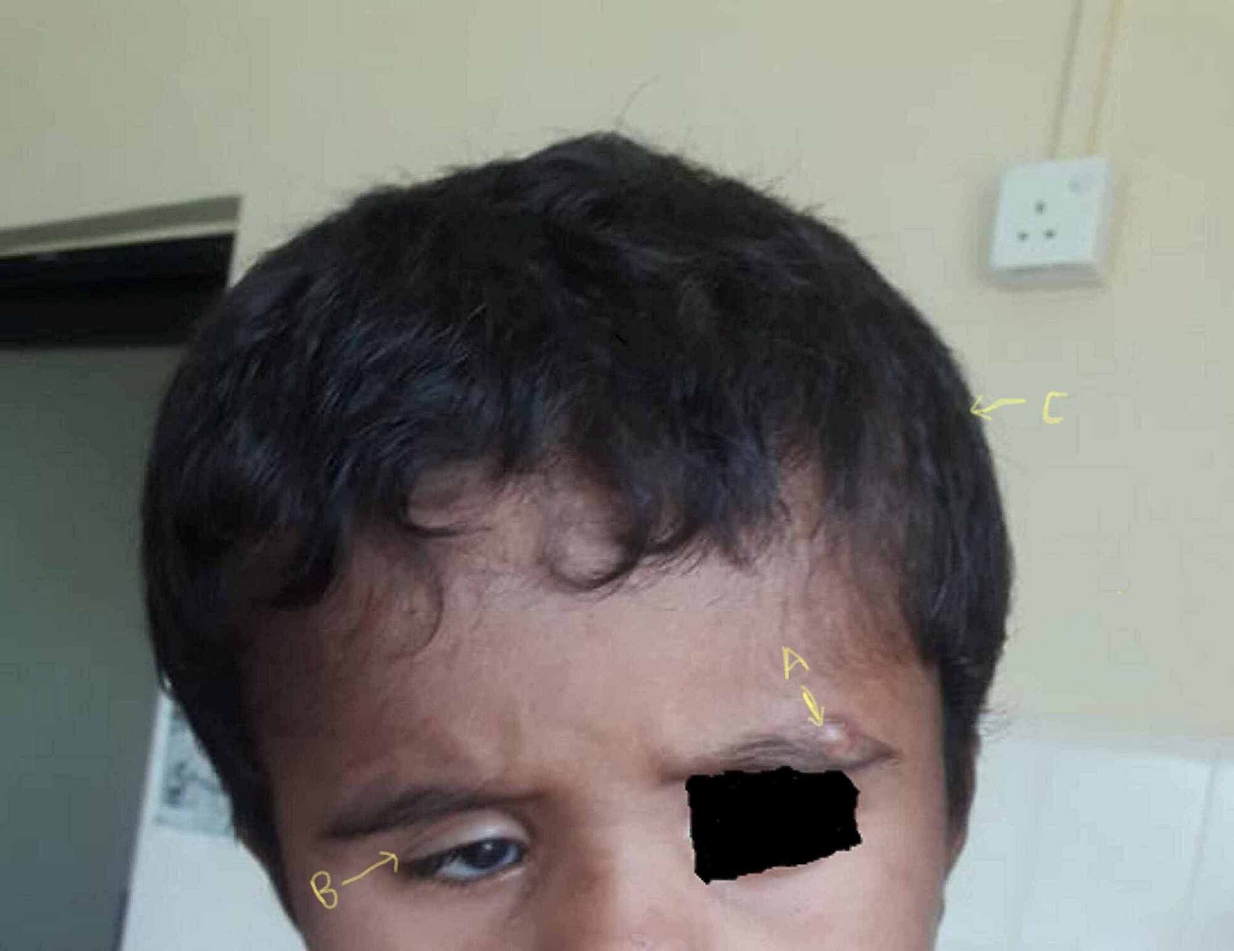
Macrocephaly (C), resolving 2.5 x 1.5 cm haemangioma over the left supra-orbital region (A), and right-side microphthalmia (B)

**Figure 2:**
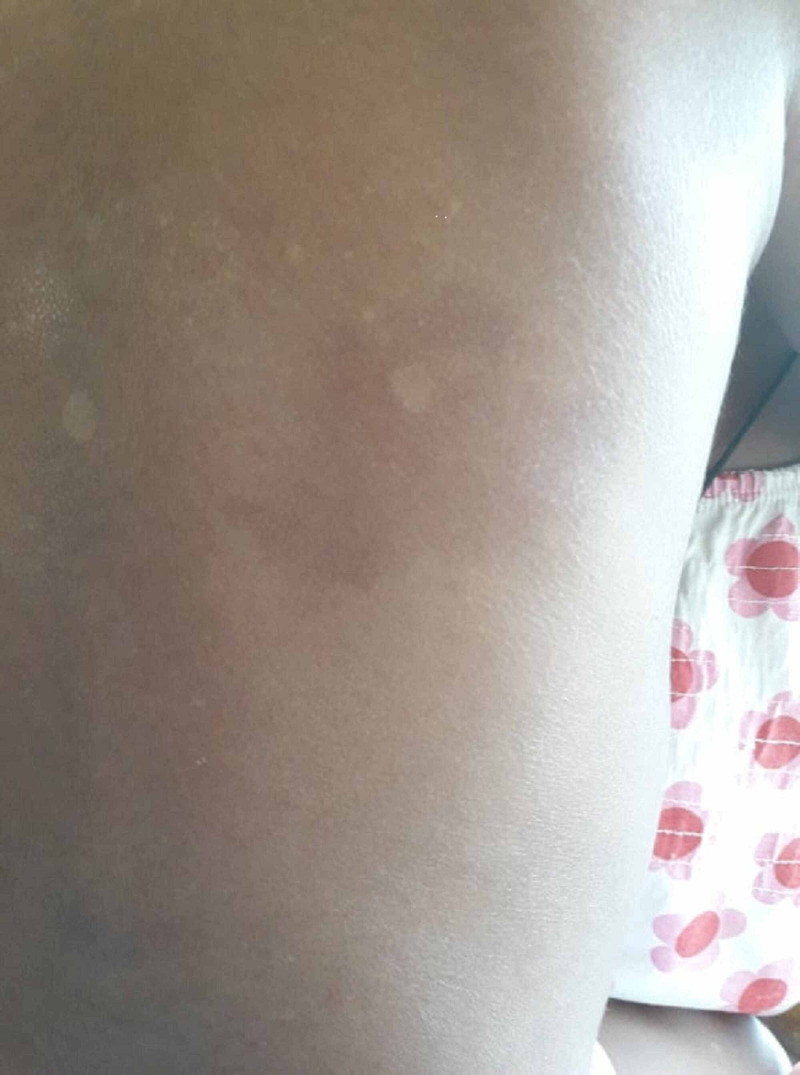
Resolving 7 x4.5 cm haemangioma below right scapular region

His growth parameters were age-appropriate: height - 98 cm (median ±1 SD), weight - 14 kg (median ±1 SD). Physical examination revealed macrocephaly (occipitofrontal circumference [OFC] - 52 cm, >90th centile), right-side microphthalmia, and several hemangiomas, including one over the left supraorbital region. Vision in the right eye was poor, with persevered normal acuity in the left eye. Neurological examination of the lower limbs and cranial nerves was normal. Four limb blood pressures were normal. He did not have midline defects such as cleft palate, sternal pits, or abdominal raphe. Figure 1 shows 7 x 4.5 cm resolving haemangioma below the right scapular region and Figure 2 shows right-side microphthalmia and 2.5 x 1.5 cm haemangioma over the left supraorbital region.

**Figure 3:**
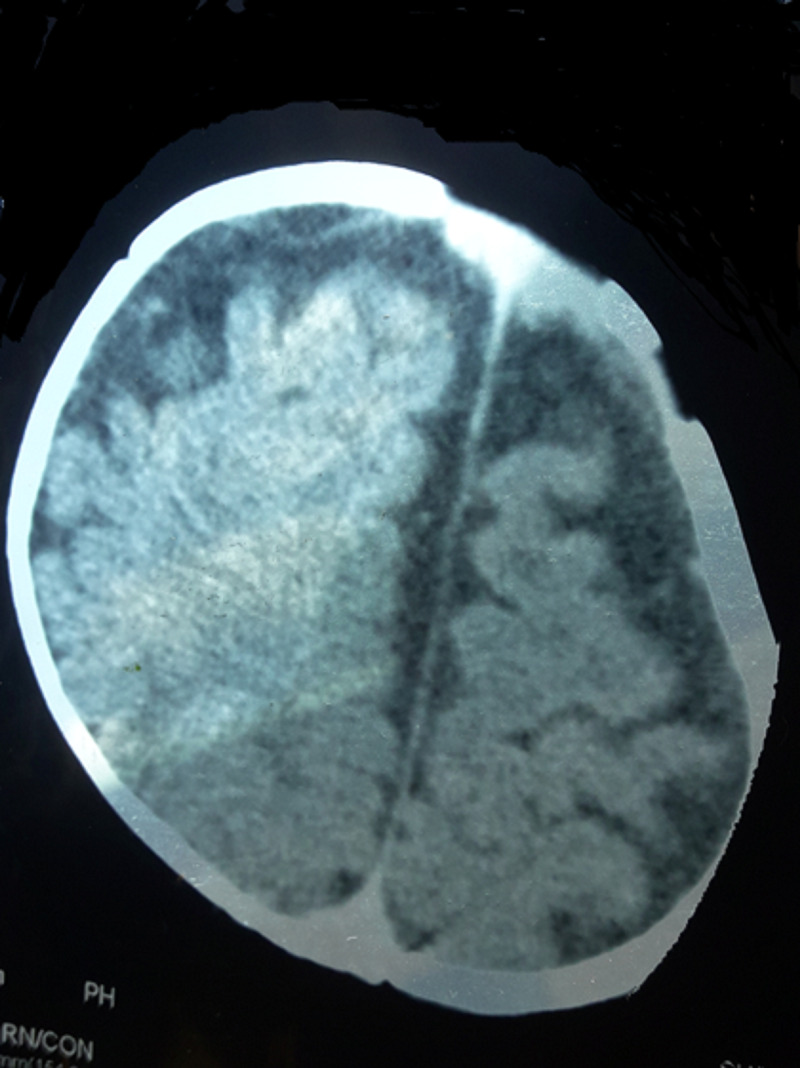
Cerebral cortical dysplasia

Computerized Tomography of the brain revealed cerebral hypoplasia. Retinal examination by an ophthalmologist revealed retinal vascular degeneration. 2D Echocardiogram revealed ostium secundum atrial septal defect. Ultrasound abdomen, thyroid functions, and hearing assessment were normal. Figure 3 shows cerebral cortical dysplasia.

Based on the revised diagnostic criteria, in addition to >5 cm haemangioma, the reported child had one major criterion and two minor criteria that confirmed the diagnosis of PHACE syndrome [4].

Propranolol was not commenced since haemangiomas were regressing in size and the potential risk of arterial disease and stroke. The child was referred to speech and communication, and occupational therapy. Follow-up was arranged with paediatrician, paediatric neurologist, and ophthalmologist.

## Discussion

The diagnosis of PHACE syndrome is based on the presence of infantile haemangioma of >5 cm in diameter with either one major criterion or two minor criteria [4]. This child, in addition to having two infantile haemangiomas (of which one regressed in size to <5 cm beyond infancy) had one major criterion (retinal vascular degeneration) and two minor criteria (microphthalmia and cerebral cortical hypoplasia) confirming the clinical diagnosis. Ventricular septal defect is recognized as a minor cardiovascular criterion [4]. However, in our child with confirmed PHACE syndrome, the only cardiac abnormality was an atrial septal defect, which is not currently recognized as a minor criterion. The detailed vascular anomalies could not be characterized due to limited imaging facilities in the reporting center. Further, the revised diagnostic criteria [4] list retinal vascular anomalies, cortical dysplasia, developmental delay, and microphthalmia as less frequent (present in <20%) features and atrial septal defect as a rare/single case report clinical feature in PHACE syndrome patients. All these clinical features were present in our child.

The mechanism underlying the origin of PHACE is believed to be due to a somatic mosaic mutation leading to altered vasculogenesis during the first trimester [5]. Since PHACE syndrome has been reported predominantly in female patients, some authors have hypothesized the possible role of X-linked inheritance and favourable skewing in mothers, but the results were not convincing [6]. Further, the males are expected to be more severely affected in X-linked disorders. However, a large study of reported patients did not reveal a higher rate and severity of complications in males, except for a slightly higher risk for structural brain abnormities [7]. Our child overall had a less severe phenotype with absent major cardiovascular anomalies, yet having cerebral dysplasia and unilateral retinal degeneration. The detailed genetic analysis of a large cohort of widely reported patients with PHACE syndrome is currently being examined [8]. The authors could not perform genetic studies on this child due to limited resources.

Management of PHACE syndrome is multifaceted. Large and problematic haemangiomas can be treated with oral propranolol. However, treatment with beta-blockers can predispose to ischaemic stroke in those with concurrent cerebral arterial anomalies [9]. In our child, both haemangiomas had spontaneously regressed in size at the time he was referred to us without diagnosis. Children with PHACE syndrome presenting with headaches need neuroimaging to rule out cerebral vasculopathies and ischaemia [4]. Specialist-led multidisciplinary follow-up, including paediatric neurologist, cardiologist, and ophthalmologist, are essential in children with neurodevelopmental, cardiac, and ocular anomalies, respectively. Children with PHACE syndrome are predisposed to endocrinological problems, such as growth and thyroid hormone deficiency, and need growth monitoring and, if required, long-term follow-up with an endocrinologist. The speech and communication delay in this child was likely due to cerebral hypoplasia, given that the hearing assessment was normal, and he needs inputs from a speech and language therapist. A dental examination is important since there have been rare reports of enamel defects. It is recommended that children with intraoral haemangiomas are referred to the orthodontist [10]. PHACE syndrome is a life-spanning disorder and, therefore, it is very important that the psychological health of children with PHACE syndrome is well attended to.

## Conclusions

This report has presented a boy in whom the rare diagnosis of PHACE syndrome was confirmed based on the revised PHACE syndrome diagnostic criteria and who had several atypical and less frequent features such as cortical dysplasia, retinal degeneration, microphthalmia, and atrial septal defect. It is pivotal that all children with PHACE syndrome receive structured health surveillance to detect comorbidities early and appropriate multidisciplinary care.
